# Vaccination with *Brucella abortus* Recombinant *In Vivo*-Induced Antigens Reduces Bacterial Load and Promotes Clearance in a Mouse Model for Infection

**DOI:** 10.1371/journal.pone.0017425

**Published:** 2011-03-11

**Authors:** Jake E. Lowry, Dale D. Isaak, Jack A. Leonhardt, Giulia Vernati, Jessie C. Pate, Gerard P. Andrews

**Affiliations:** 1 Department of Veterinary Sciences, University of Wyoming, Laramie, Wyoming, United States of America; 2 Department of Molecular Biology, University of Wyoming, Laramie, Wyoming, United States of America; 3 Professional Veterinary Medicine Program, College of Veterinary Medicine & Biomedical Sciences, Colorado State University, Fort Collins, Colorado, United States of America; University of Massachusetts Medical Center, United States of America

## Abstract

Current vaccines used for the prevention of brucellosis are ineffective in inducing protective immunity in animals that are chronically infected with *Brucella abortus*, such as elk. Using a gene discovery approach, in vivo-induced antigen technology (IVIAT) on *B. abortus*, we previously identified ten loci that encode products up-regulated during infection in elk and consequently may play a role in virulence. In our present study, five of the loci (D15, 0187, VirJ, Mdh, AfuA) were selected for further characterization and compared with three additional antigens with virulence potential (Hia, PrpA, MltA). All eight genes were PCR-amplified from *B. abortus* and cloned into *E. coli*. The recombinant products were then expressed, purified, adjuvanted, and delivered subcutaneously to BALB/c mice. After primary immunization and two boosts, mice were challenged i.p. with 5×10^4^ CFU of *B. abortus* strain 19. Spleens from challenged animals were harvested and bacterial loads determined by colony count at various time points. While vaccination with four of the eight individual proteins appeared to have some effect on clearance kinetics, mice vaccinated with recombinant Mdh displayed the most significant reduction in bacterial colonization. Furthermore, mice immunized with Mdh maintained higher levels of IFN-γ in spleens compared to other treatment groups. Collectively, our in vivo data gathered from the S19 murine colonization model suggest that vaccination with at least three of the IVIAT antigens conferred an enhanced ability of the host to respond to infection, reinforcing the utility of this methodology for the identification of potential vaccine candidates against brucellosis. Mechanisms for immunity to one protein, Mdh, require further in vitro exploration and evaluation against wild-type *B. abortus* challenge in mice, as well as other hosts. Additional studies are being undertaken to clarify the role of Mdh and other IVI antigens in *B. abortus* virulence and induction of protective immunity.

## Introduction

Brucellosis continues to be problematic to the agriculture industry world-wide, including the U.S. Furthermore, several *Brucella* spp. have been classed as category B threat list agents with the potential for use as bioterrorism weapons. Efforts to develop an effective, stable, and non-reactogenic vaccine against brucellosis have been ongoing in several laboratories, and the use of a live, attenuated platform has become the established benchmark through the use of the *B. abortus* rough strain RB51 [1]. Although moderate efficacy against *Brucella*-induced fetal abortions in domestic livestock (cattle) has been reported [2], acceptable levels of protection following immunization with RB51 has yet to be demonstrated in wildlife such as elk [3], [4], [5], [6], and in the case of bison, results have been conflicting in terms of the vaccine's reactogenicity [7], [8], [9], [10], [11]. The exact nature of the attenuation of RB51 is also unclear, although it's rough LPS phenotype is due to at least one lesion in O-side chain biosynthesis loci [1]. A more systematic approach to the induction of active protective immunity against brucellosis has been undertaken by some laboratories through the development of subunit vaccines [12], [13], [14], [15], [16], [17]. To date, the degree of success in protecting with such vaccines depends on the ability of the candidate to drive immunity towards a Th1-type response, emphasizing the need to identify and characterize *Brucella* antigens which present T-cell epitopes to the host [18]. Despite the efforts to identify components for a next-generation subunit vaccine, formulations using recombinant *Brucella* antigens have not been thoroughly assessed for immunogenicity/efficacy. The discovery of additional *Brucella* virulence factors thus may facilitate the development of a more efficacious, less reactogenic, acellular product that may either be used as a stand-alone vaccine or used to augment primary immunization with the existing live, attenuated platform. As an example of the latter strategy, enhanced efficacy in the mouse model has been reported by over-expressing *Brucella* superoxide dismutase (SOD) in RB51 or complementing the strain's rough LPS phenotype with the O-side chain biosynthesis locus, *wboA* [19].

We previously applied the gene discovery methodology, known as in vivo-induced antigen technology (IVIAT), to identify *B. abortus* virulence genes up-regulated during infection in elk (*Cervis elaphus*), and as a result have identified ten loci with gene products potentially important to survival of the pathogen in this host [20]. Furthermore, the conserved nature of most of these gene products has led us to extend our hypothesis - that they also may be requisite virulence effectors in other *Brucella* susceptible hosts. As a preliminary approach to confirming our hypothesis, we have selected five of these in vivo-induced (IVI) products for further characterization in a surrogate murine model for *B. abortus* colonization: a conserved outer membrane protein, D15; a gluconeogenic enzyme, malate dehydrogenase (Mdh); a periplasmic component of an ABC transport system, AfuA; a component of the Type-IV secretion system (T4SS) VirJ; and a lipoprotein of unknown function BAB1_0187 (referred to as 0187). We also targeted three additional conserved gene products based on high amino acid sequence similarity with antigens identified through *Yersinia pestis* IVIAT and previous reports of a role in *Brucella* pathogenesis [21], [22]: proline epimerase (PrpA), an auto-secreting (Type-V) surface antigen, Hia, and a soluble lytic transglycosylase, MltE.

## Results

### S19 Infection Kinetics

To establish the colonization kinetics of *B. abortus* S19 in BALB/c mice in our laboratory, thirty naive animals were infected with S19 at 5×10^4^ CFU and five animals sacrificed at 7, 14, 21, 28, 42, and 70 days post-infection. Bacterial loads in spleens peaked in two weeks at 8×10^7^ CFU before gradually declining to 6×10^3^ CFU in 6 weeks. At 10 weeks post infection, organisms were still able to be cultured from spleens in 60% of the animals. Also, splenomegaly was observed in S19-infected mice, peaking between 14 and 21 days post-infection, and declining by day 28 (data not shown).

### S19 challenge after vaccination with recombinant IVI products

In the first experiment, thirty mice were vaccinated with purified Mdh, MltE, or adjuvant-only. After hyper-immunization with the recombinant proteins, the sera were assessed for antibody. End-point titers from mice immunized with the recombinant protein ranged from a 1∶1000 to 1∶5000. Serum from mice receiving alhydrogel alone was non-reactive against any of the proteins.

After challenge, bacterial loads in the spleens were determined at 7, 14, 21, and 28 days post-infection. As shown in Table 1, mice vaccinated with Mdh showed a markedly significant decrease in bacterial colonization at 14 dpi, providing 2.75 log units of clearance and 2 log units of clearance at 21 days post-infection compared to adjuvant-only treated animals (p<0.001). Bacterial loads measured in MltE-immunized mice were no different than the adjuvant-only controls (not shown). Mdh-immunized animals also displayed extended splenomegaly which remained elevated relative to the adjuvant-only animals at 28 days post-infection (data not shown). By 42 days, the Mdh-immunized animals had completely cleared the infection, while S19 was still cultured from spleens of the adjuvant-only mice at the same time point (data not shown).

**Table 1:** Reduction of bacterial load and more rapid clearance in a murine colonization model by immunization with recombinant *B. abortus* Mdh.

Time, Post-infection (days)	Adjuvant Only	Mdh	p value	Log reduction
**0**	-	-	**-**	**-**
**7**	2.51×10^6^(+/− 3.34×10^5^)	**8.05×10^5^**(+/− 5.62×10^5^)	**<0.05**	**0.55**
**14**	9.94×10^7^(+/− 2.17×10^7^)	**2.43×10^5^**(+/− 1.12×10^5^)	**<0.001**	**2.75**
**21**	2.64×10^6^(+/− 8.57×10^5^)	**3.73×10^4^**(+/− 2.08×10^4^)	**<0.001**	**2.09**
**28**	1.38×105(+/− 8.37×10^4^)	**1.29×10^4^**(+/− 1.00×10^4^)	**<0.05**	**1.09**

In a second experiment, fifteen mice each received alhydrogel only, AfuA, Hia or D15 recombinant proteins adsorbed to the adjuvant. Mice were primed and boosted twice, and serum was collected to assay for antibody titers. Reactivity of the immune sera to all three antigens was detectable out to 1∶1000 for Hia and 1∶5000 for D15 and AfuA (comparable to that of Mdh immunized mice). The animals were challenged as before and bacterial loads in the spleens assessed at 14, 21, and 28 days post-infection. As shown in Table 2, in contrast to Mdh, there was no significant difference at two weeks between the adjuvant-only and antigen-immunized animal groups, however at three weeks post-infection all three experimental groups differed significantly from the adjuvant-only group with D15 showing the most pronounced effect of 1.41 log units of clearance (p<0.001). As observed with Mdh-immunized animals, splenomegaly remained consistently elevated in all test groups at 28 days post- infection relative to the adjuvant-only control animals (data not shown).

**Table 2:** Reduction of bacterial load in a murine colonization model by immunization with recombinant *B. abortus* AfuA, D15, or Hia.

Time (days)Post-infection	AdjuvantOnly	AfuA	D15	Hia	p value	Logreduction
**0**	-	-	-	-		
**7**	2.51×10^6^	ND	ND	ND		
	(+/− 3.34×10^5^)					
**14**	9.94×10^7^	2.89×10^7^	4.40×10^7^	1.88×10^7^		
	(+/− 2.17×10^7^)	(+/− 1.78×10^7^)	(+/− 2.23×10^7^)	(+/− 7.02×10^6^)		
**21**	2.64×10^6^	**6.53×10^4^**	**1.23×10^5^**	**4.20×10^4^**	**<0.001**	**1.41**
	(+/− 8.57×10^5^)	(+/− 5.38×10^4^)	(+/− 8.05×10^4^)	(+/− 2.81×10^4^)	**(D15)**	
**28**	1.38×10^5^	**1.59×10^4^**	**1.50×10^4^**	**2.89×10^4^**	**<0.05**	**1.91**
	(+/− 8.37×10^4^)	(+/− 9.31×10^3^)	(+/− 9.08×10^3^)	(+/− 1.27×10^4^)	**(Hia)**	

A third iteration was then conducted to evaluate VirJ, 0187, and PrpA. Following the same methods as above, antibody titers were determined to be >1∶5000 (VirJ, PrpA and 0187). Mice were challenged with S19 and splenic colony counts were performed as described previously at 14 and 21 days post-infection. No significant difference was found between adjuvant-only animals and those receiving any of the three antigens (data not shown), although all immune animals displayed some degree of splenomegaly.

### Mouse cytokine response to challenge with S19

Levels of IL-12p70, IL-4 and IFN-γ in the splenic homogenates were quantified from the five selected animal groups immunized with antigens which had an effect on bacterial load and/or clearance rate. No detectable IL-12p70 or IL-4 groups immunized with AfuA, Hia, D15, or adjuvant alone was observed. IL-4 was, however, detected in Mdh-vaccinated mice, although at low levels (data not shown).

**Figure 1:**
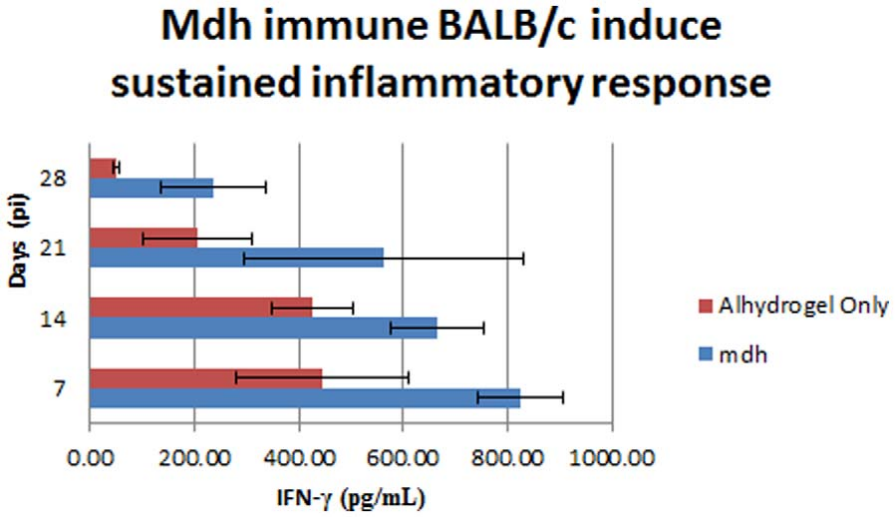
IFN-γ response in Mdh-immunized BALB/c mice after S19 challenge. Spleen homogenates from the five mice from each time point (7,14,21 and 28 dpi) sacrificed during the challenge studies were evaluated for IFN-γ production.

IFN-γ was next assessed in vaccinated mice after challenge. As shown in Figure 1, at all sampling times, mice vaccinated with Mdh showed significantly higher levels of this cytokine (p<0.05), compared to the mice receiving AfuA, D15, or alhydrogel alone. By 21 and 28 days post-infection, IFN-γ levels among all groups had declined with the exception of the Mdh immune group, in which IFN-γ remained significantly elevated (p<0.05; Figure 1).

### In Vivo assessment of IVI gene up-regulation during S19 infection in mice

**Figure 2:**
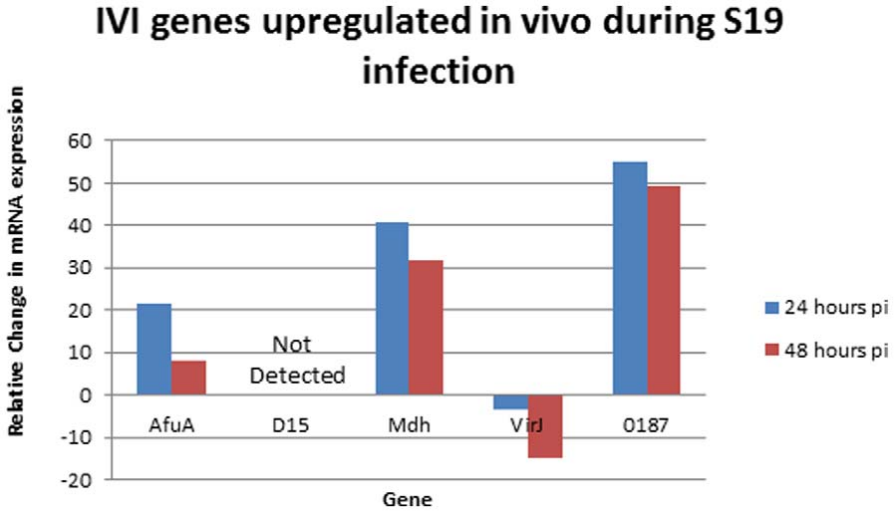
IVI genes upregulated in vivo during S19 infection. Average Fold change of bacterial mRNA isolated from five mice infected with S19 at each time point compared to in vitro-grown B. abortus S19.

“Short-unique” regions of selected IVI genes identified through IVIAT from elk infected with wild-type *B. abortus* were selected for construction of RT-PCR primers. Ten BALB/c mice were subsequently infected with S19, five of which were splenectomised at 24 and 48 hours post-infection, and bacterial mRNA isolated. Additionally, S19 was grown to mid-log phase in vitro and mRNA extracted for comparison. Quantitative analysis of cDNA showed up-regulation during both 24 and 48 hours post-infection of *afuA*, *mdh*, and 0187 (Figure 2). In contrast, D15 mRNA was not detected in either the in vitro or in vivo samples, even after performing several different nested RT-PCR reactions (data not shown). While, *prpA* and *mltE* were not examined, Hia-encoding transcript was expressed at equivalent levels in vitro and during infection (data not shown).

## Discussion

The use of a murine model for the characterization of *B. abortus* IVI proteins as potential protective antigens is likely not the optimal animal model for simulation of the disease state in the natural host species. Consequently, behavior of recombinant antigens and/or mutants may be different in mice than the target species. Despite this drawback, some mouse strains have been shown to be highly sensitive to the pathogen, and have been used to study *B. abortus* pathogenesis and evaluate vaccine candidates for the past two decades [23], [24], [25], [26], [27], [28]. Furthermore, studies with S19 in pregnant BALB/c mice reported an identical pathology, placentitis and septic fetal death, as with wild-type *B. abortus* infection, further supporting the applicability of this model to the simulation of disease in other host species [28], [29]. The benefit of using S19 instead of wild-type *B. abortus* is reduced cost and safety, since BSL-3 small animal containment facilities are not required.

Vaccination with purified *B. abortus* Mdh resulted in significantly reduced colonization and more rapid clearance of S19 in the BALB/c mouse. Interestingly, Mdh was the only recombinant protein of the five antigens examined which facilitated some level of clearance that elicited a significant IFN-γ response, a cytokine critical for the activation of macrophages and a requisite for controlling *Brucella* infections [30]. It is therefore likely that prolonged elevated levels of IFN-γ in mice vaccinated with Mdh contribute to the reduction in the colonization by S19 in these immune animals. The nature of Mdh-induced immune-mediated enhanced clearance is unclear at present, however, it is possible that an auxiliary virulence function of the enzyme may be neutralized by a robust immune response directed toward it. The presence of IL-4 and absence of IL-12 also suggest that mice vaccinated with Mdh induce a Th2-biased response leading to clearance immunity mediated by antibody. In fact, a search for putative T- and B-cell epitopes across the amino acid sequence of the protein revealed only the latter (data not shown). This hypothesis may seem contrary to the traditional notion that only a Th1-biased response can reduce intracellular bacterial load in the *Brucella*-infected host. Indeed, previous experiments with other facultative intracellular pathogens such as *Yersinia pestis* have demonstrated that antibody alone can confer protection against challenge [31]. In the case of *B. abortus*, an “optimized” Th2 response might also contribute significantly to clearing infection.

Although, immunity to AfuA and D15 failed to elicit more rapid clearance of S19, bacterial loads were significantly reduced in animals immunized with either of these two proteins. In contrast to AfuA and Mdh, D15 expression was not evident early in S19 colonization, thus D15 may be relevant at a later stage of infection in the mouse. Curiously, in our previous study [20], we failed to detect antibody to Mdh and D15 in S19-immunized elk, indicating that both proteins may be regulated differently in cervids.

Hia was not identified as an IVI gene in our initial study [20], and consistent with this finding, was subsequently was found to be constitutively expressed based on our mRNA analysis (data not shown). Our selection of this gene product was based on similar homology to *Y. pestis* protein and previous reports in the literature of involvement as a virulence factor [32]. This type-V auto-secreted antigen, induced a greater level of extended splenomegaly compared to the other recombinant proteins, including Mdh. However, the increased inflammation did not correlate with heightened production of IFN-γ (data not shown). This observation suggests that perhaps immunity to Hia increases the inflammatory response during infection by mechanisms not related to IFN-γ, such as TNF-α or IL-1. Although immunization with Hia reduced bacterial load in our model comparable to D15 and AfuA, it failed to induce more rapid clearance.

As with MltE, 0187, PrpA, and VirJ failed to reduce bacterial load and/or alter clearance kinetics compared to adjuvant-only controls. 0187 is a putative lipoprotein that shares significant homology with the well characterized BA14K protein. Previous studies have demonstrated that BA14K was able to induce a Th1 response and induce IL-12 secretion [33]. We were unable to express 0187 from *B. abortus* as a full length protein but were able to stably express a truncated form shortened by 27 amino acids from the n-terminus (data not shown). It is possible that this truncation could have resulted in conformation changes leading to an inability to induce clearance immunity.

The proline epimerase, PrpA, was described as a B-cell polyclonal activator and inducer of IL-10 [22], suggesting that immunity to this protein may promote clearance in our model based on the pivotal role IL-10 plays in *Brucella* pathogenesis [30], [34]. Mice immunized with PrpA during early infection actually had splenic counts higher than animals immunized with adjuvant alone. It is possible that a secondary exposure to PrpA results in host immune dysregulation early during the course of infection.

The data from our previous study strongly suggested that the Type-IV secretion system (T4SS) accessory protein, VirJ, is up-regulated during wild type *B. abortus* infection in elk. Animals immunized with S19 however did not generate a humoral response to the protein (unpublished data), which suggests a difference in the way this secreton is utilized by S19 in cervids. Consistent with this finding, in the S19 murine colonization model, VirJ appeared to be slightly down-regulated at least during early stages of infection. A BLAST analysis upstream and downstream of the VirJ-encoding locus revealed no differences between S19, 2308 or 9-941 (Wyoming strain) DNA sequences, suggesting the involvement of a distal regulatory element(s) in controlling expression of this protein in S19. The function of VirJ, as assessed in other pathogens, is suspected to be as a periplasmic chaperone responsible for assisting substrates in associating with a “pusher” pilus before translocation through the T4SS, typically thought to be required for full virulence in this pathogen [35]. In this regard, we intend to re-evaluate VirJ in a *B. abortus* 2308 challenge model.

Data from our model system is in agreement with that previously published for S19 colonization kinetics in BALB/c mice [23]. Our vaccination efforts with a single recombinant protein, Mdh, coincide with previously reported data on mice vaccinated with RB51 in terms of the subsequent cytokine responses post-challenge [36]. IFN-γ levels peak between 6 and 7 days then begin to slowly decline, albeit remaining sustained for weeks [36]. RB51 vaccinates also lack significant production IL-12p70 or high levels of IL-4 upon challenge. [36]. This observation could be peculiar to BALB/c mice, which tend to be more biased towards humoral responses [1], [30], [36]. As predicted, our S19 data shows that a pro-inflammatory response is suppressed in naïve animals and behaves similarly to strain 2308 in this respect [30]. This observation suggests that a shift in cytokine production levels could be important in providing a more efficacious immune response to brucellosis.

Taken together, these data suggest the potential for use of the gluconeogenic enzyme, malate dehydrogenase, as a recombinant subunit vaccine candidate for brucellosis. AfuA, D15, and/or Hia may also represent promising subunit vaccine candidates when used together and/or in combination with Mdh. In this regard, we plan to evaluate such antigen cocktails in the near future, using wild type field isolates of *B. abortus* in mice and other host species.

## Methods

### Bacterial strains and growth conditions

*Brucella abortus* S19, was kindly provided by the Colorado Serum Company (Denver, CO), and was used exclusively for this study in the mouse colonization/infection model. Brain-heart infusion broth cultures were typically grown overnight at 37°C, serially diluted after three washes in sterile PBS, followed by plating to determine a viable cell count correlate with optical density at 600 nm.

### In vivo gene expression, RNA extraction, and RT-PCR

Ten BALB/c mice were infected with 1×10^7^ cfu of *B. abortus* S19 i.p. Mice were splenectomized and tissues stored in RNAlater™ (Ambion, Austin, TX). Tissues were homogenized and RNA isolated with the RiboPure-Bacteria™ Kit (Ambion, Austin, TX). Isolated RNA was transcribed to cDNA using RETROscript™ (Ambion, Austin, TX) and cDNA targets amplified by *PfuTurbo*™ DNA Polymerase (Stratagene, La Jolla, CA) in a one-step reaction. Amplification of a segment of the 16S subunit of *B. abortus* S19 was used as a positive control; negative controls were included for each gene and contained all the reaction components except reverse transcriptase. In addition a negative control was employed which lacked RNA template to confirm the absence of DNA contamination in the reaction. Concentration of PCR product in gel bands was assessed using Quantity One™ 4.6 software (Bio-Rad, Hercules, CA).

### Plasmid construction, recombinant protein expression, and purification

Selected IVI genes were amplified in their entirety from the *B. abortus* RB51 genome by PCR, using PfuUltra Master Mix (Stratagene, La Jolla, CA), inserted into the pET-46 Ek/LIC™ system (EMD Biosciences, La Jolla, CA), and transformed into *E. coli* NovaBlue cells (EMD Biosciences, La Jolla, CA). The recombinant plasmid constructs were then purified and the insert sequence confirmed by PCR and sequencing. The recombinant plasmids were re-transformed into *E. coli* Rosetta-2[DE3] cells (EMD Biosciences, La Jolla, CA) and induced to express under 0.5 mM IPTG at 30°C. Verification of expression of recombinant products was performed by total crude protein resolution on SDS-PAGE followed by Western blot analysis using a His-tagged Monoclonal antibody (EMD Biosciences, La Jolla, CA). Ten mls of 0.5 mM IPTG-induced cultures of recombinant *E. coli* strains were treated with BugBuster HT (EMD Biosciences, La Jolla, CA) and soluble (AfuA, Mdh, MltE, 0187) and insoluble (D15, VirJ, Hia) fractions containing recombinant histidine-tagged fusion proteins purified by the HisMag™ Purification Kit (EMD Biosciences, La Jolla, CA). Insoluble fractions containing D15, VirJ, and Hia were purified under 8 M Urea, followed by dialysis against PBS. The proteins were run on a SDS-PAGE gel to confirm purity and quantified by spectral absorption at 280 nm and BCA Lowry (Pierce Chemical).

### Subunit vaccine preparation

Purified proteins were mixed with a 1∶7 dilution of aluminum hydroxide adjuvant (Alhydrogel™; Superfos, Denmark) in PBS and adsorbed overnight at 4°C at a concentration of 150 µg/mL.

### Animal studies

All animals utilized in this study were cared for according to strict adherence to the Policies and Regulations established by the US Public Health Service “Humane Care and Use of Laboratory Animals” and an approved animal protocol from the University of Wyoming Institutional Animal Care and Use Committee (IACUC) (DHHS Assurance #A3216-1).

Ten to 30 six-week old, female BALB/c mice received 30 µg of recombinant protein subcutaneously in 200 µL of adjuvant at one site. Additional control mice were treated with adjuvant only in the same manner. Immunization regimen consisted of a prime and two boosts, 21 days apart. Retro-orbital bleeds were performed to assess antibody titers by Western blot. Animals were challenged i.p. with 5×10^4^ organisms of *B. abortus* S19 at 14 days after the second boost. Five mice from each group were sacrificed at specific time points. Spleens were removed, weighed, homogenized, used to determine whole organ bacterial load following serial dilution of the homogenates in 1X Sterile PBS and plating on blood agar. The remaining homogenates were stored at −40°C for cytokine analysis.

### Cytokine analysis

Supernatants from spleen homogenates were used in QuantiKine™ ELISA Assays (R & D Systems, Minneapolis, MN) to quantify IL-12p70, IL-4, and IFN-γ cytokine levels in the spleen.

### Statistics

All statistical analysis was performed with the software package SAS 9.1 Enterprise™ (SAS Corporation, Cary, NC). ANOVA was used to compare means of groups and Least Significant Difference (LSD) was used to determine mean separations between the groups. α = 0.05; p values are listed in text.
